# Artificial intelligence to improve the detection and risk stratification of acute pulmonary embolism (AID-PE): protocol for a pragmatic quasi-experimental comparator study

**DOI:** 10.1136/bmjopen-2025-111826

**Published:** 2026-02-12

**Authors:** Samuel George Sinclair Gunning, Joseph Page, Jennifer Rossdale, Pia Frances Pemberton Charters, Benjamin Hudson, Stephen Lyen, Robert Mackenzie Ross, Annette Seatter, Jonathan W Bartlett, Lisa Austin, Gareth Myring, Hugh McLeod, Paul Mitchell, Darryl Stimpson, Andrew Cookson, Jay Suntharalingam, Jonathan Carl Luis Rodrigues

**Affiliations:** 1 Department of Research and Development, Royal United Hospitals Bath NHS Foundation Trust, Bath, UK; 2 Department of Respiratory, Royal United Hospitals Bath NHS Foundation Trust, Bath, UK; 3 Department of Radiology, Royal United Hospitals Bath NHS Foundation Trust, Bath, UK; 4 Department of Medical Statistics, London School of Hygiene and Tropical Medicine, London, UK; 5 Department for Health, University of Bath, Bath, UK; 6 The National Institute for Health and Care Research Applied Research Collaboration West (NIHR ARC West), University Hospitals Bristol and Weston NHS Foundation Trust, Bristol, UK; 7 Health Economics and Health Policy at Bristol (HEHP@Bristol), University of Bristol, Bristol, UK; 8 Bath Pulmonary Hypertension Patient Advisory Group, Royal United Hospitals Bath NHS Foundation Trust, Bath, UK; 9 Department of Mechanical Engineering, University of Bath, Bath, England, UK; 10 Department of Life Sciences, University of Bath, Bath, UK; 11 Department of Health, University of Bath, Bath, UK

**Keywords:** Artificial Intelligence, Computed tomography, Health Care Costs, Thromboembolism, Pulmonary Disease

## Abstract

**Introduction:**

Pulmonary embolism (PE) is a potentially fatal condition requiring timely diagnosis and treatment. CT pulmonary angiography (CTPA) is the gold standard for diagnosis and indicates PE severity through radiological markers of right heart strain. However, accurate interpretation and communication of these findings is often suboptimal in real-world practice. Artificial intelligence (AI) could alleviate pressure on radiology services by supporting PE identification, risk stratification and worklist prioritisation. Before widespread adoption, AI tools must be rigorously validated for diagnostic accuracy, safety and clinical impact.

**Methods and analysis:**

This pragmatic single-centre, non-randomised quasi-experimental study will evaluate the diagnostic accuracy, feasibility, and clinical-cost impact of AI-assisted PE detection and risk stratification using AIDOC and IMBIO software. We will recruit two consecutive cohorts of adult patients undergoing CTPAs for suspected PE: a comparator cohort (12 months pre-AI implementation) and an intervention cohort (12 months post-AI implementation). AI will be applied retrospectively to the comparator cohort, while in the intervention cohort, radiologists will have contemporaneous access to the AI’s interpretation of CTPA images.

A subset of retrospective scans, both PE-positive and PE-negative, will undergo expert thoracic radiologist review to establish a reference standard. Data on patient demographics, clinical management and outcomes will be collected. Clinical management pathways and patient outcomes will be compared between cohorts to assess AI’s influence on acute PE management. Health economic modelling will assess the cost-effectiveness of integrating AI technology within the diagnostic workflow of acute PE.

**Ethics and dissemination:**

This study was approved by the UK Healthcare Research authority (IRAS 311735, 10 May 2023). Ethical approval was granted by West of Scotland Research Ethics Service (23/WS/0067, 3 May 2023). Results will be shared with stakeholders, presented at national and international conferences, and published in open-access peer-reviewed journals.

**Trial registration number:**

NCT06093217.

**STRENGTHS AND LIMITATIONS OF THIS STUDY**

- This quasi-experimental study will use retrospective and prospective cohorts to evaluate the real-world clinical and economic impact on patients with suspected acute pulmonary embolus (PE) of integrating artificial intelligence (AI) for detection and risk stratification of PE into radiology workflows.
- The clinical ground truth will be re-established in a retrospective cohort via expert thoracic radiologist review.
- Comprehensive data collection, capturing a range of clinical and radiological endpoints, will enable robust, multidimensional analyses of the clinical and economic impact of implementing AI.
- As a single-centre study, findings may support future national, multi-centre research validating AI for acute pulmonary embolism detection and risk stratification in broader populations.

## Introduction

### Background

 Pulmonary embolism (PE) is a potentially fatal condition that may cause right ventricular failure and death if not recognised and treated promptly. In the UK, PE accounts for almost 70 000 hospital presentations and 37 000 hospital admissions per year.^1^ This places a substantial financial and operational burden on the NHS. Although it is difficult to know precise cost estimates, the total cost burden for venous thromboembolism is estimated at £640 million per year.^2^ Furthermore, the incidence of PE is rising.^3^,^5^ This trend can be partially explained by the increasing availability and improved quality of diagnostic imaging. However, demographic and clinical factors, such as increased survival among patients with major prothrombotic comorbidities and obesity, are also likely to contribute to this increase.^6^

Although falling, mortality from PE remains significant. It accounts for up to 6.5 deaths per 100 000 population per year.^6^ The reduction in mortality is attributed to improved interventions, including direct oral anticoagulants, better management guidelines^7^ and increased diagnosis of smaller, subsegmental emboli.^8^ However, despite advances in PE detection and clear national guidance on acute PE treatment, risk stratification and management are still performed poorly.^9^

Radiological imaging plays a critical role in the PE pathway. Multi-detector CT pulmonary angiography (CTPA) is considered the gold standard imaging modality for PE diagnosis. CTPA can also provide important, prognostically relevant information on right heart strain (RHS).^10 11^ This includes reversal of the right ventricular to left ventricular ratio (RV:LV), interventricular septal straightening and contrast reflux in the inferior vena cava. These indirect markers can inform risk stratification and subsequent clinical medical management.

The NHS Long Term Plan outlines the importance of innovative, affordable technologies to improve diagnosis, risk assessment and treatment of respiratory conditions, including PE.^12^ Achieving this depends on accurate image interpretation and timely communication of findings. However, national audits, including the National Confidential Enquiry into Patient Outcome and Death, have highlighted deficiencies in PE reporting and downstream clinical.^9^

### Challenges in current practice

While CTPA is widely available and has high diagnostic accuracy for detecting acute PE, growing demand places increasing pressure on radiology departments.^13^ These pressures, particularly during times of service strain, can contribute to delays in scan interpretation and increase the likelihood of fatigue-related diagnostic errors.^14^ Additionally, interobserver variability remains a persistent challenge. Non-specialist radiologists may differ in their ability to confidently identify prognostically significant features of radiological RHS despite their clinical relevance. Consequently, radiological reporting of the RV:LV ratio is often inconsistent or omitted altogether.^9 15^ This variability undermines the standardisation of acute PE care and may lead to missed opportunities for both escalation of treatment in high-risk patients and timely discharge in those identified as low risk.

### Artificial intelligence in radiology

Artificial intelligence (AI) assisted image interpretation of CTPA scans offers a potential solution to these challenges. AI algorithms may enhance reporting accuracy, reduce inter-reporter variability, prioritise high-risk cases and support workflow efficiency for acute PE. AI tools have demonstrated strong performance compared with acute reporting radiologists in acute PE identification with reported sensitivities and specificities as high as 97% and 100% respectively.^16 17^ AI algorithms have also been shown to outperform radiologists in the detection of PE and improve reporting times through case prioritisation.^16 18^

Although AI algorithms for RHS are less well established, early studies suggest they can reliably quantify RV:LV ratio.^10^ Further validation of these algorithms in prospective populations and within the UK is required. Furthermore, few studies have evaluated the clinical utility or cost-effectiveness of integrating such AI software into radiology workflows in real-world settings.

### Study aims and objectives

This study aims to evaluate the real-world clinical and financial impact of integrating AI algorithms into radiological workflows for the assessment and management of acute PE within a single-centre, district general hospital. The study will evaluate the implementation of two commercially available, Food and Drug Administration (FDA) approved AI tools designed to assist radiologists with acute PE detection and quantification of RHS.

The study will examine whether AI integration can improve accuracy of PE diagnosis, enhance risk stratification through RHS quantification, and support downstream clinical decision-making. The study will compare the outcomes observed between a pre-AI and post-AI implementation cohort.

The research team hypothesises that AI-assisted interpretation of CTPA will lead to earlier identification of high-risk patients, minimise delays in critical decision-making and contribute to more consistent application of evidence-based PE management strategies.

## Methods: participants, interventions and outcomes

This study protocol was designed in accordance with Standard Protocol Items: Recommendations for Interventional Trials guidelines.^19^

### Study design

This pragmatic, single-centre, non-randomised, quasi-experimental cohort study will evaluate clinical and radiological outcomes in two distinct patient cohorts. The comparator cohort is defined as *usual care*; general radiologist reporting of CTPA. This cohort will consist of patients recruited retrospectively, prior to implementation of the AI. The intervention cohort is defined as *integrated AI technology AND usual care*; general radiologist reporting of CTPA with access to AI technology for acute PE detection. This cohort will consist of patients recruited prospectively, following the implementation of the AI.

**Figure 1:**
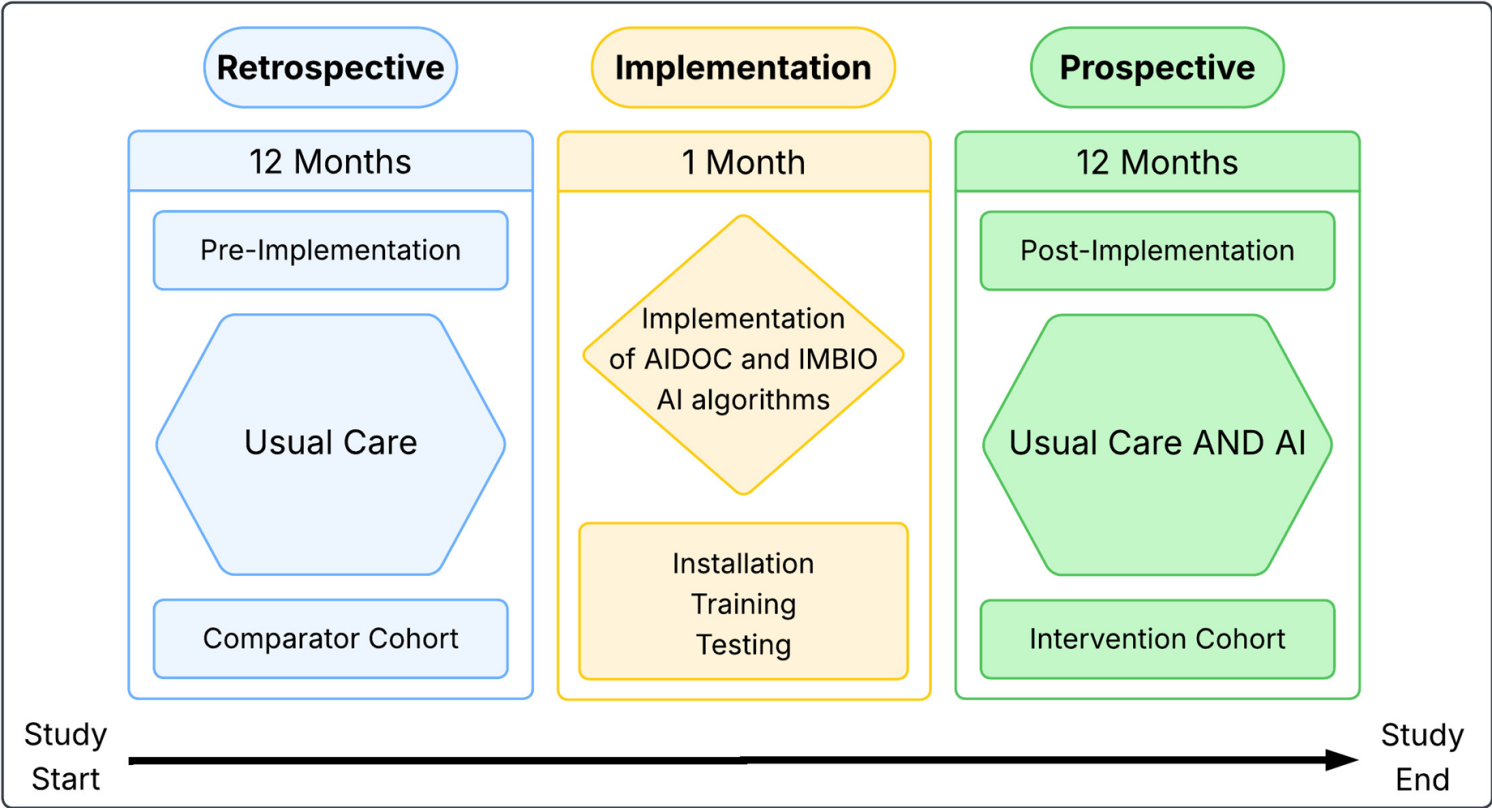
Study schedule and recruitment. AI, artificial intelligence.

The study will consist of three distinct phases. The pre-implementation and post-implementation phases will derive the comparator and intervention cohorts, respectively. The implementation phase will ensure installation, testing and appropriate training of the AI technology. Figure 1 outlines the study structure.

### Study aim

To evaluate the diagnostic performance of integrated AI for the detection of PE and RHS on CTPA and establish the clinical and economic impact of AI assistance on the diagnosis, risk stratification and downstream management of patients presenting with suspected acute PE.

### Study objectives

The retrospective cohort analysis will assess the diagnostic performance of AI in detecting acute PE and quantifying associated RHS. The study will compare AI outputs against routine clinical radiology reports as well as a redefined ground truth through expert thoracic radiologist review of a subset of retrospective CTPAs. The prospective cohort analysis will assess the real-world impact of AI integration on clinical practice, including risk stratification, treatment decisions and patient outcomes. The study will compare concordance of acute PE risk stratification and patient management with local protocols and European Society of Cardiology guidelines.^11^ The study will also assess the clinical-cost impact of AI integration into radiology workflows.

Finally, the study will explore the perspective of key stakeholders, including radiologists and referring clinicians, on the implementation and use of AI technology before, during and after deployment.

### Study population and setting

Study participants will be recruited from consecutive, clinically indicated CTPAs performed for the assessment of acute PE at Royal United Hospitals Bath NHS Foundation Trust in England. Scans from both inpatient and outpatient settings will be included. Ethical approval for participant inclusion has been granted by West of Scotland Research Ethics Service (23/WS/0067, 3 May 2023).

The pre-implementation (comparator) cohort will be retrospectively identified over a 12-month period preceding AI deployment. Following a 1 month implementation phase, the post-implementation (intervention) cohort will be prospectively recruited over the subsequent 12 months. Prospective recruitment will complete on 7th January 2025.

All participants will meet the following eligibility criteria:

#### Inclusion criteria

- Adults over 18 years old.
- CTPA performed for the clinical assessment of acute PE.

#### Exclusion criteria

Participants will not be eligible for inclusion in the study if any of the following are true:

- Aged under 18 years old.
- Registered with the national opt-out scheme for research.
- CTPA performed for reasons other than the assessment of acute PE.
- CTPA reported by external radiologist.
- CTPA scan which is incomplete or discontinued.

Patients whose CTPA was reported by an external radiologist will be excluded due to difficulty confirming the reporting radiologist’s access to existing AI software. Patients will also be excluded if the clinical indication for CTPA is unrelated to acute PE. The AI software is solely licenced for the assessment of acute PE. No substantial amendments have been made to the eligibility criteria since study inception.

### Sample size

As a pragmatic real-world quasi-experimental assessment of AI integration, formal power calculations were not feasible. Based on historic imaging volumes, it is anticipated that approximately 3500 eligible CTPAs will be identified across the 24-month recruitment period and that the prevalence of acute PE will be between 12% and 15%.

### Screening and consent

The research team will establish the comparator and intervention cohorts by screening all consecutively reported CTPAs identified through the radiology information system (RIS). Consent was explicitly discussed with patient and public involvement (PPI) participants during the design and planning of the study. PPI participants were supportive of automated AI assessment at the time of CTPA imaging and did not express concerns over its use as a decision-support tool. The ethics committee waived the need for formal patient consent given that all AI outputs will be reviewed and verified by a human radiologist prior to inclusion in any clinical reports. This approach was deemed acceptable due to the absence of direct patient risk.

### Participant data

Clinical, radiological and patient-related outcomes will be extracted for each participant using a combination of electronic and paper-based systems. Primary data sources will include electronic health records, integrated care records, RIS and physical patient notes where relevant. Data extraction will be conducted by trained members of the research team using a pre-specified case report form (CRF) to ensure consistency.

The following data types will be collected for each participant:

1. Demographics: age and sex.
2. Anthropometrics: height, weight and body mass index.
3. Referral context: date and time, outpatient or inpatient location.
4. Admission/discharge details: admission and discharge date and time.
5. Comorbidities: structured past medical history entries and coded diagnoses.
6. Physiological parameters: vital signs closest to the time of CTPA request (ie, heart rate, blood pressure, oxygen saturation, respiratory rate).
7. Biochemistry markers: including, but not limited to, d-dimer, troponin, brain natriuretic peptide.
8. Imaging history: chest radiography, echocardiography, lower extremity limb Doppler ultrasound.
9. Medication history: including, but not limited to, anticoagulants, antiplatelets and time to anticoagulation initiation.
10. Patient management details: time of first clinical review, location of ongoing care and referrals to respiratory, thrombosis and haematology clinics.
11. Acute PE severity assessment: simplified pulmonary embolism severity index (sPESI) and the Geneva Score calculated based on recorded clinical parameters.^20 21^
12. Clinical outcomes up to 12 months after their CT data: including, but not limited to, intensive care admission, haemodynamic instability, thrombolysis administration, re-presentation to the emergency department, re-admission to the hospital, major and minor bleeding events and all-cause mortality.

### Interventions

#### CT report interpretation

All radiology reports for consecutive CTPAs in the comparator and intervention cohorts will be reviewed by a respiratory registrar with 8 years’ clinical experience. The reports will be classified as either positive or negative for acute PE. In cases of uncertainty, adjudication will be performed by a second senior clinician.

The same registrar and a second clinician, blinded to each other’s decisions, will independently assess the reports for radiologist assessment of RHS. Reports will be categorised as either positive, negative or ‘no comment’ for RHS. Discrepancies will be resolved through discussion and, if required, arbitration from a third clinician. This approach will establish a consistent comparator for evaluating AI outputs.

#### AI technology and application

The AI solutions under evaluation are provided by AIDOC, who offer two FDA approved AI solutions for acute PE detection and assessment of the RV:LV ratio. The latter AI tool is created by IMBIO for quantification of RHS. The AI solutions are both CE-marked and operate within UK information governance standards.

**Figure 2:**
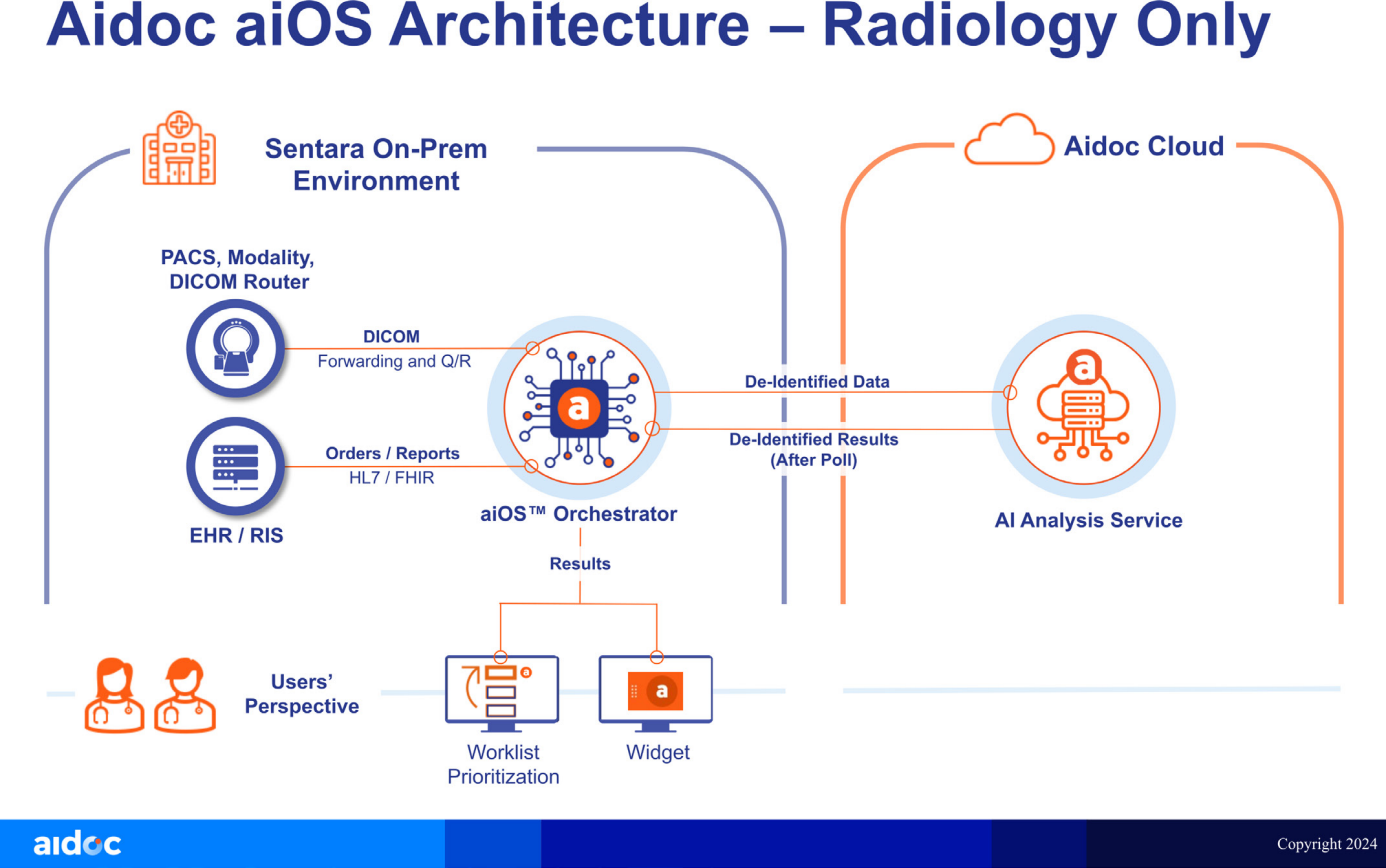
Schematic demonstrating the integration of AI solutions into the normal clinical workflow (AIDOC Copyright 2024 - Proprietary and Confidential). AI, artificial intelligence; EHR, electronic health record; PACS, picture archiving and communication system; RIS, radiology information system.

The AI solution uses Digital Imaging and Communications in Medicine (DICOM) data that is sent to the on-premises AIDOC Orchestrator from one or more DICOM sources (picture archiving and communication system (PACS), vendor neutral archive, DICOM router or modality scanners). The Orchestrator uses AI-based computer vision to identify the anatomy in the image series and determines which AI algorithms can be run. It then de-identifies the DICOM before sending it to the AIDOC cloud environment for analysis. No patient identifiable data leaves the customer network (ie, the Trust), and no communication is initiated from the cloud. The Orchestrator polls the cloud for AI results, which are typically available within 2–6 min. Once available, the Orchestrator downloads the AI results via a secure tunnel connection and distributes them accordingly (typically via prioritisation alerts in the AIDOC Desktop App and via radiology worklist prioritisation). Figure 2 illustrates the data flow between the Trust, cloud environment and Orchestrator.

##### Comparator cohort: usual care

In the comparator cohort, AI will be applied retrospectively and will not influence clinical decision-making at the time of initial scan reporting. This cohort will represent usual care and inform the accuracy and potential clinical utility of integrated AI technology for acute PE.

##### Reference standard

Three cardiothoracic radiologists will independently review a 4 month subset of consecutive CTPA images within the retrospective comparator cohort. They will initially be blinded to the retrospective application of the AI output and initial radiologist report to establish the ‘ground truth’ radiological findings of the CT scans. In the event of acute PE uncertainty, a second cardiothoracic radiologist will be sought for arbitration. This dataset will be used as the reference standard.

##### Intervention cohort: AI technology plus usual care

AI tools will be integrated prospectively into radiology workflows within the intervention cohort. The AI technology features a priority flagging system which alerts radiologists to suspected PE cases. This prioritisation process does not re-order the RIS queue or auto-populate any radiology report. Instead, the AI software generates key-slice images and RV:LV ratio overlays that are directly accessible within the PACS and AIDOC Desktop App. This allows the reporting radiologist to consider the AI output at their discretion when reporting. Verification of the final radiology report will remain the responsibility of the reporting radiologist.

For CTPAs not analysed by the AI technology due to technical issues, standard reporting procedures will be followed.

During the intervention phase, radiologists will have the option to provide direct feedback on AI outputs via the priority flagging system, including free-text commentary if desired. All cases in which there is a discrepancy between the AI output and radiology report will be reviewed by the research team as part of a predefined sub-analysis. This will allow assessment of radiologist engagement with the AI outputs, its diagnostic accuracy and clinical relevance in routine practice.

##### Unequivocal PE

The research team will only intervene if the AI software identifies an unequivocal acute PE that appears to have been missed or not reported in the intervention cohort. These cases will be escalated to a thoracic radiologist for urgent review to ensure appropriate treatment. A study-specific standard operating procedure has been designed to manage this process while maintaining patient safety.

##### Clinical management

Royal United Hospitals Bath NHS Foundation Trust does not currently have a dedicated Pulmonary Embolism Response Team or onsite percutaneous thrombectomy service. There are no plans to implement either of these during the study period. Consequently, acute PE management follows standard clinical pathways.

The AI acts as an adjunct to routine clinical care, supporting detection of acute PE and automated assessment of RHS. We therefore anticipate any outcome differences will reflect how radiologists incorporate AI-derived findings into their reports and how clinicians act on them, rather than the influence of specialist services or advanced interventional pathways.

## Methods: data collection, management and analysis

### Data collection and management

For each eligible participant, a structured CRF will be completed to capture relevant data. Only the minimum necessary data required to address the study objectives will be collected. Participants will be assigned a unique study identification code to pseudonymise their data. A cross-referencing list linking identifiers to study codes will be stored securely on a password-protected NHS computer database. All research records will be retained for 5 years, after which they will be securely destroyed.^22^

For the comparator cohort, anonymised CTPA images will be transferred for off-site analysis at AIDOC’s core laboratory (Tel-Aviv, Israel). Prior to image transfer, all imaging will be anonymised in accordance with the Royal United Hospitals Bath NHS Foundation Trust’s information governance protocols to ensure data confidentiality.

In the intervention cohort, AI solutions will be embedded within existing PACS workflows. AIDOC and IMBIO servers will be installed locally, facilitating real-time ‘live’ AI analysis of CTPAs. System integration will be conducted in collaboration with radiology information technology and information governance teams.

### Statistical analysis

#### Clinical and radiological outcomes

Our primary outcome will be the proportion of patient decisions made in line with evidence-based best practice guidelines after introducing AI technology within CTPA reporting. Secondary outcomes relate to acute PE detection, RV:LV ratio quantification and clinical and operational endpoints including mortality, length of stay, readmission, follow-up and cost to NHS (table 1).

**Table 1:** Primary, secondary and additional outcomes

Outcome measure	Measure description
Primary outcome measure	
Proportion of patient decisions made in line with evidence-based best practice guidelines after introducing AI technology within CTPA reporting	Comparison before and after AI introduction
Secondary outcome measures	
Rate of acute PE detection with AI technology	True positives and true negatives
Rate of discordant acute PE cases	False positive and false negative rate with acute PE detection
AI failure rate for acute PE detection	Proportion of scans unable to be interpreted by AI despite suitable CTPA acquisition
Rate of RV:LV detection with AI technology	True positive and true negative
Rate of discordant RV:LV detection	False positive and false negative
Failure rate for automated RV:LV ratio	Proportion of scans unable to calculate automated RV:LV ratio despite suitable CTPA acquisition
30-day mortality	Patient mortality (death) at 30-days post-PE diagnosis. Comparison before and after AI introduction
12-month mortality	Patient mortality (death) at 12 months post-PE diagnosis. Comparison before and after AI introduction
Hospital admission and bed days for acute PE	Comparison before and after AI introduction
Time to anticoagulation in PE cases	Comparison before and after AI introduction
Time from CTPA to discharge	Comparison before and after AI introduction
PE risk stratification rates (low, intermediate low, intermediate high and high risk)	Comparison before and after AI introduction
Cost to NHS for acute PE	Comparison before and after AI introduction
End-user (clinician and radiologist) acceptability of AI technology	Quantified metrics from a non-validated questionnaire to evaluate end-use experience of integrated AI radiology reporting
Referral rates to outpatient follow-up (respiratory, thrombosis, haematology)	Comparison before and after AI introduction
Diagnostic rate of chronic thromboembolic pulmonary hypertension	Comparison before and after AI introduction
Additional outcome measures	
Exploratory outcomes	Given the exploratory nature of this observational non-randomised feasibility study, there may be patterns/outcomes which emerge/develop during the study period. The investigators will report on any patterns that may emerge following the introduction of AI reporting

AI, artificial intelligence; CTPA, CT pulmonary angiography; PE, pulmonary embolism; RV:LV, right ventricular to left ventricular ratio.

Descriptive statistics will summarise participant baseline characteristics and comorbidities, separately in the comparator and intervention cohorts. Our primary analyses will consist of comparisons between the comparator and intervention cohorts, with continuous outcomes analysed using t-tests and categorical outcomes using χ^2^ tests. To account for and disentangle any possible underlying time trends from the effects of AI introduction, regression models will be used to compare outcomes. These will include covariates that allow for a linear trend in the pre-implementation phase, a step change at the point of AI implementation, and a modification to the linear trend following this.

Kaplan-Meier survival curves will quantify differences between time-to-event outcomes in the two cohorts. Cox regression models will also be used to summarise the difference between cohorts. If feasible, regression models will be adjusted for known predictors of outcome from both Geneva and sPESI scoring systems.^20 21^ Missing values will be handled by performing each analysis excluding patients who have relevant data missing for the variables concerned. Statistical significance will be set according to p value <0.05.

#### Artificial intelligence accuracy

We will evaluate AI performance against both the original radiology reports and expert thoracic radiologist reviews, which will serve as the clinical reference standard. Agreement between modalities will be assessed using Cohen’s kappa and measures of diagnostic test performance.

The research team will also review all PE-positive and discrepant cases to evaluate the potential for the AI software to act as a clinical safety net for the detection of acute PE.

#### Health economics

An exploratory decision analysis model will be developed. The model will assess the cost-effectiveness of integrating AI technology within the diagnostic workflow for acute PE. The model includes a decision tree component and Markov cohort. The decision tree will model the diagnosis, treatment and clinical outcomes for acute PE. Evidence for the decision tree will be derived from the comparator cohort. The Markov cohort will evaluate the lifetime costs and outcomes for acute PE. Evidence for these costs will be drawn from the literature on the consequences of the post-pulmonary syndrome. Unit costs associated with radiological imaging, hospitalisation and treatment will be extracted from NHS reference costs, unit costs of health and social care and the hospital finance team.

Uncertainty in parameter inputs will be accounted for in the analysis by including parametric distributions at each point estimate. In cases where the evidence is not sufficiently detailed, expert opinion will be used to estimate the most plausible distribution. Threshold analysis will be used to address the commercially sensitive nature of the cost of adopting the AI technology. Results from the model will be reported as incremental cost-effectiveness ratios and expected net monetary benefit. Cost-effectiveness acceptability curves will show the impact of uncertainty in the cost-effectiveness.

## Trial management and study governance

The trial management group (TMG) will be led by the chief investigator and includes the full research team. The TMG is responsible for the daily running of the study.

The conduct of the research will be monitored by an independent trial steering group (TSG). The TSG will consist of an independent cardiothoracic radiologist, pulmonary vascular physician, health economist and patient representative. The TSG will focus on the progress of the study, patient safety and provide independent expert advice when required. The chief investigator and trial manager will attend all TSG meetings throughout the study.

Adverse events (AEs) and serious adverse events are anticipated to be minimal due to mitigating factors within the study design. TMG will discuss any AEs with the TSG and report these to the sponsor and research ethics committee.

## Patient and public involvement

PPI was obtained during the design of the study. This included patients with lived experience of acute PE and chronic thromboembolic pulmonary hypertension. The study incorporates a patient advisory group (PAG), who will meet with the research team throughout the study and support activities to assist dissemination of the study findings. PAG members will also be invited to attend TMG meetings.

## Ethics and dissemination

Ethical approval was granted by West of Scotland Research Ethics Service (23/WS/0067, 3 May 2023). Study approval was granted by the Healthcare Research Authority (IRAS 311735, 10 May 2023). Study results will be discussed with the research team, its collaborators and other key stakeholders. The results will be disclosed in a summarised form to the public with support from the PAG. The research team will aim to present the study findings at national and international conferences relevant to the application of AI in healthcare. The research team aims to publish the results in open-access peer-reviewed journals. A summarisation of the project and outcomes will be distributed to the public with support from the PAG using video media.

## Data statement

The data collected during this study cannot be shared publicly for the privacy of individuals that participate in the study. The data may be shared on reasonable request to the corresponding author.
