# Supplementary material for: Artificial intelligence to improve the detection and risk stratification of acute pulmonary embolism (AID-PE): protocol for a pragmatic quasi-experimental comparator study
Source: BMJ Open. 2026 Feb 12;16(2):e111826. doi: 10.1136/bmjopen-2025-111826 (PMC12911836; doi:10.1136/bmjopen-2025-111826)
Supplement: Supplementary file 1 [file bmjopen-2025-111826.reviewer_comments.pdf]

PEER REVIEW HISTORY

BMJ Open publishes all reviews undertaken for accepted manuscripts. Reviewers are asked to complete a checklist review form and are provided with free text boxes to elaborate on their assessment. These free text comments are reproduced below.

ARTICLE DETAILS

Title (Provisional)

Artificial Intelligence to improve the Detection and risk stratification of acute Pulmonary Embolism (AID-PE): protocol for a pragmatic quasi-experimental comparator study

Authors

Gunning, Samuel George Sinclair; Page, Joseph; Rossdale, Jennifer; Charters, Pia Frances Pemberton; Hudson, Benjamin; Lyen, Stephen; Mackenzie Ross, Robert; Seatter, Annette; Bartlett, Jonathan W; Austin, Lisa; Myring, Gareth; McLeod, Hugh; Mitchell , Paul; Stimpson, Darryl; Cookson, Andrew; Suntharalingam, Jay; Rodrigues, Jonathan Carl Luis

---

VERSION 1 - REVIEW

**Reviewer**	**1**
**Name**	**Kojodjojo, Pipin**
**Affiliation**	**Asian Heart and Vascular Centre**
**Date**	**18-Oct-2025**
**COI**	**None**

The authors report the study protoccol for the AID-PE trial.

Comments:

1. Has the recruitment for prospective phase completed? In the manuscript, the expected completion date of the study is not stated

2. Incomplete sentence:

The NHS Long Term Plan outlines the importance of innovative, affordable technologies to improve diagnosis, risk assessment, and treatment of respiratory conditions, including [12].

3. What happens in terms of clinical management after the report is generated? I would anticipate that time from ordering scan > report will be significantly shortened. The pre-order phase should be the same. There are significant clinical changes to PE management with the introduction of PERT and percutaneous thrombectomy treatments. How can you be sure that any improvement in survival / long term outcomes is due to the AI rather than

improved clinical management, expedited treatment, increased experience of the treating team?

VERSION 1 - AUTHOR RESPONSE

Suggestion	Response
**Reviewer #1**	
Has the recruitment for prospective phase completed? In the manuscript, the expected completion date of the study is not stated	Thank you for this comment. Prospective recruitment is complete — final completion date was 7 January 2025. A sentence to this effect has been added to the manuscript.
Incomplete sentence: The NHS Long Term Plan outlines the importance of innovative, affordable technologies to improve diagnosis, risk assessment, and treatment of respiratory conditions, including [12].	Thank you for highlighting this omission. This has now been corrected.
What happens in terms of clinical management after the report is generated? I would anticipate that time from ordering scan > report will be significantly shortened. The pre-order phase should be the same. There are significant clinical changes to PE management with the introduction of PERT and percutaneous thrombectomy treatments. How can you be sure that any improvement in survival / long term outcomes is due to the AI rather than improved clinical management, expedited treatment, increased experience of the treating team?	Thank you for this question. Following generation of a CTPA report, individual clinicians are expected to read and act on findings in a timely fashion. The RUH does not currently have a dedicated PERT team or onsite percutaneous thrombectomy service and consequently acute PE management follows standard clinical pathways. The AI system acts as an adjunct to routine clinical care, offering earlier detection of acute PE and automated assessment of right heart strain. Therefore, any outcome differences reflect how radiologists incorporate AI-derived findings within their reports and how clinicians act on them, rather than the influence of specialist services or advanced interventional pathways. A paragraph outlining clinical management pathways has been added to the manuscript.
